# Reversal of Strain State in a Mott Insulator Thin Film by Controlling Substrate Morphology

**DOI:** 10.1002/smll.74217

**Published:** 2026-06-23

**Authors:** Reetendra Singh, Abhishek Rakshit, Galit Atiya, Mikhail Kalina, Yaron Kauffmann, Yoav Kalcheim

**Affiliations:** ^1^ Department of Materials Science and Engineering Technion‐Israel Institute of Technology Haifa Israel

**Keywords:** metal‐Insulator transition, Mott insulator, phase transition, strain engineering

## Abstract

The phase diagram of *V*_2_ *O*_3_ contains two insulating phases and one metallic phase with distinct lattice structures whose stability is highly strain sensitive. In epitaxial thin films, strain is typically controlled through lattice mismatch with the substrate. Here, we show that substrate morphology itself can become a key control parameter by enabling thermal expansion mismatch to dominate the strain state. We investigate *V*_2_ *O*_3_ films grown on sapphire, where lattice mismatch induces compressive strain while thermal expansion mismatch produces tensile strain. By modifying the sapphire surface morphology through annealing, which generates either atomically flat or stepped surfaces, the compressive strain can be partially relaxed. This relaxation allows the tensile thermal strain to overcome the lattice‐mismatch contribution, resulting in either strongly compressive or strongly tensile strain states in otherwise nominally identical films. High‐resolution STEM reveals crystallographic defects forming near substrate steps, identifying the microscopic origin of the enhanced relaxation. These morphology‐controlled strain states strongly affect the electronic properties: compressive strain suppresses the metal‐insulator transition, whereas tensile strain stabilizes insulating phases at all temperatures, producing resistivity changes spanning many orders of magnitude. Our results establish substrate morphology as a powerful route for strain engineering in correlated oxide thin films.

## Graphical abstract

Minute changes in substrate morphology, such as the formation of atomic steps, are found to strongly influence strain in thin films. The steps are induced by substrate annealing and promote strain relaxation through the formation of defects during growth. Along with thermal expansion mismatch, modifying the substrate morphology offers a simple and effective tuning knob for electronic properties in strongly correlated materials.

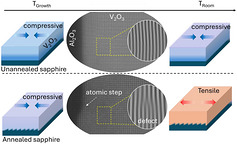

## 1 Introduction

V_2_O_3_ is a prototypical Mott system and one of the most widely studied quantum materials, owing to its rich phase diagram and a first‐order metal‐insulator transition (MIT) that involves a strong interplay of structural, electronic, magnetic, and orbital degrees of freedom [1, 2, 3, 4]. At room temperature and ambient pressure, bulk V_2_O_3_ is a paramagnetic metal (PM) with a corundum structure. When cooled, it undergoes a transition to a monoclinic antiferromagnetic insulator (AFI) at a transition temperature of ∼160 K [4]. In bulk V_2_O_3_, the resistivity changes across the MIT by more than seven orders of magnitude [1]. While the pronounced sensitivity of V_2_O_3_ and other strongly correlated electron systems to external stimuli underpins their potential for applications in memory devices, resistive RAM selectors, optical switches, and neuromorphic systems, their MIT innot yet fully understood [5, 6, 7, 8, 9]. Previous reports suggested that the properties of Mott insulator films may be influenced by growth temperature, oxygen partial pressure, substrate orientations, and their treatment [10, 11, 12, 13, 14, 15, 16]. Strain is a key factor influencing the MIT in V_2_O_3_ films, as it directly modifies orbital overlap and lattice distortions that govern the electronic and magnetic states [17, 18, 19, 20, 21, 22, 23, 24, 25, 26, 27]. Both defects and substrate‐induced strain play a decisive role in tuning the transition of V_2_O_3_ and other Mott insulators [22, 23, 24, 25, 26, 27], highlighting the importance of strain engineering in controlling MIT behavior. Pofelski et al. demonstrated that V_2_O_3_ films of varying thicknesses grown on c‐sapphire exhibit a strongly suppressed MIT, with the extent of suppression depending on the film thickness [28]. Additionally, a wide range of strain control in V_2_O_3_ and (V_0.985_Cr_0.015_)_2_O_3_ films on *c*‐sapphire was realized by inserting (Cr_1‐ᵧ_Fe_ᵧ_)_2_O_3_/Cr_2_O_3_ buffer layers and systematically varying the Fe content [29].

Despite this large body of work, several crucial aspects of strain engineering in V_2_O_3_ are not well understood—namely the interplay between different mechanisms for strain formation such as lattice mismatch, thermal expansion mismatch, and strain relaxation by defect formation. Indeed, it is often found that the characteristics of the transition, such as the critical temperature, thermal transition width, and hysteresis, vary widely depending on the choice of substrate, but also for seemingly similar substrates. To understand the complete phenomenology observed in V_2_O_3_ thin films, we focus on sapphire substrates, which are the most widely used since the two materials are isostructural, allowing for heteroepitaxial growth [10, 11, 12, 28, 29, 30, 31, 32]. The lattice constants of sapphire are smaller than those of V_2_O_3_ – by 7.8% in the *c*‐axis and 4.1% perpendicular to it. This may result in large compressive epitaxial strain in the V_2_O_3_ films whose magnitude depends on strain relaxation mechanisms taking place during growth. An additional source of strain is the substrate/film thermal expansion mismatch. Comparing lattice constants of V_2_O_3_ and sapphire between typical growth temperatures (∼1000 K) and room temperature, one finds a differential lattice constant mismatch between the substrate and film which would result in ∼0.9% compressive strain in the *c*‐axis and ∼0.74% tensile perpendicular to the *c*‐axis (assuming perfect clamping) [33, 34]. Therefore, when V_2_O_3_ films are grown on *c*‐plane oriented sapphire, the lattice constant mismatch tends to compress the films in the plane, while the thermal expansion mismatch has an opposite effect. Due to the high sensitivity of Mott insulators to strain, understanding the role of these competing effects is of paramount importance. Here we show that by controlling the substrate morphology, the degree of strain relaxation at the growth temperature may be controlled, thereby diminishing the compressive strain component and driving the film into a tensile strain regime upon cooling. It is found that a simple annealing pretreatment of the substrate allows us to tune the properties of V_2_O_3_ from a state of nearly complete MIT suppression and metallic behavior at low temperatures to a fully developed resistive transition and a paramagnetic insulating (PI) phase at room temperature. By imaging the defects that form in the V_2_O_3_ films with high‐resolution STEM and correlating them with substrate morphology, we shed light on the mechanism by which strain is induced in V_2_O_3_ films. This method of strain engineering can be used to drive V_2_O_3_ into different regimes of the phase diagram, induce an insulating state above room temperature, and vary resistance in different films by more than 7 orders of magnitude. The strain control mechanism, which is elucidated here, may be applicable to many other systems where epitaxial and thermal strains play a significant role.

## 2 Results

To confirm growth quality, four 100 nm heteroepitaxial thin films of V_2_O_3_ were grown on sapphire substrates with different orientations, using RF magnetron sputtering from a stoichiometric V_2_O_3_ target. The films were grown in a 5 mTorr Ar atmosphere at a substrate temperature of 750°C, followed by thermal quenching at a rate of ∼90°C/min [14]. Resistance vs. temperature measurements reveal that the films grown on sapphire with orientations other than (0001) show a pronounced MIT with a resistance change of 6‐7 orders of magnitude (Figure S1) [21]. However, the film grown on (0001)‐sapphire exhibits a strongly suppressed MIT, since the c‐plane lattice vectors are aligned parallel to the substrate surface and thus constrained by it (Figure S2) [26, 28]. The confinement restricts the in‐plane lattice expansion needed for the corundum‐to‐monoclinic phase transition, which in turn suppresses the MIT. In this study, we examined the effect of substrate morphology on the strain induced in films grown on (0001)‐sapphire, the mechanism by which the substrate morphology affects the strain, and the resulting film transport properties.

**FIGURE 1:**
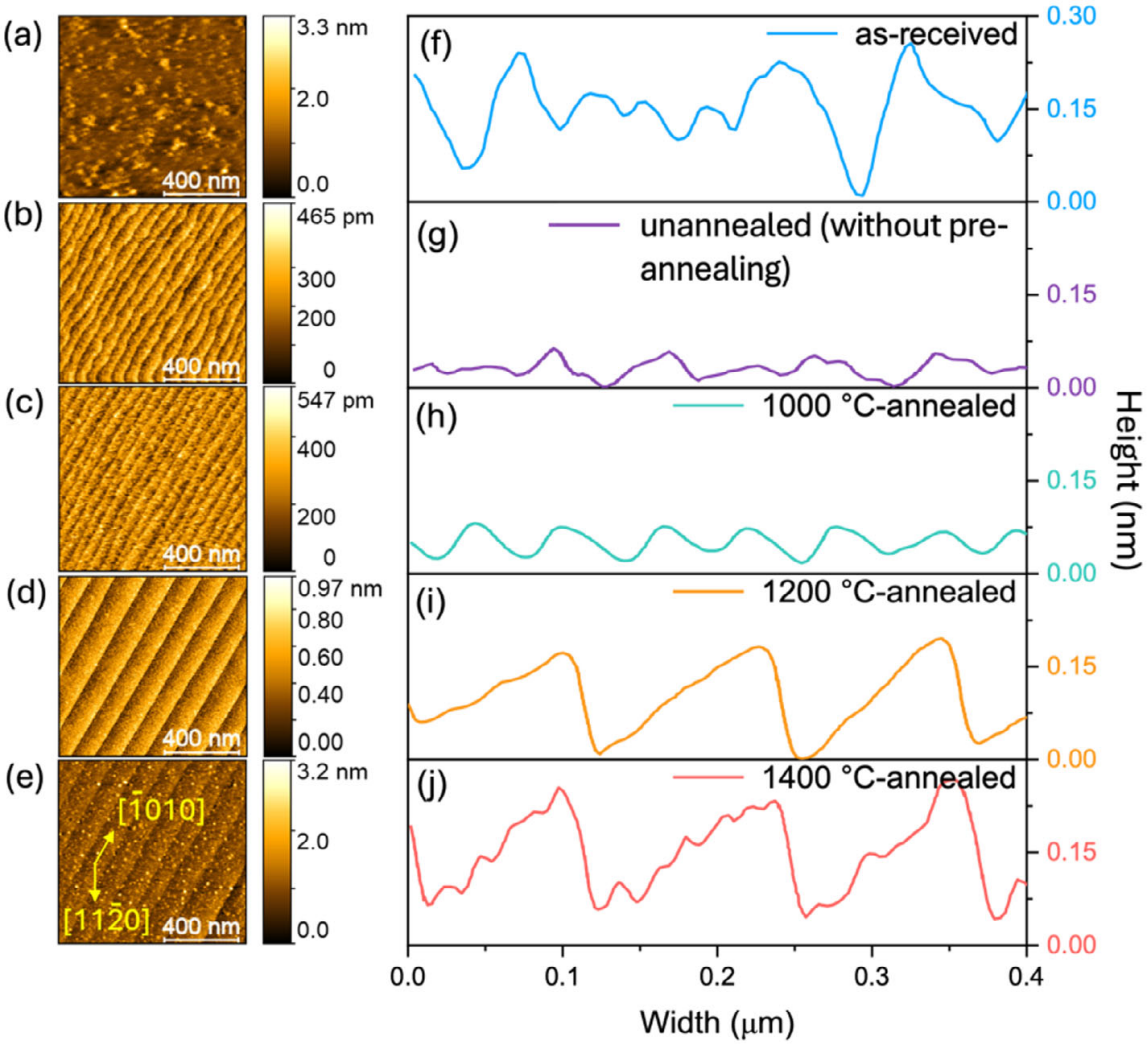
Effect of annealing on sapphire substrate morphology: (a–e) AFM images of as‐received (0001)‐sapphire showing no atomic steps, whereas annealed substrates exhibit well‐defined atomic steps. (f–j) height profile across the steps taken from the topographic images (a–e); The step heights and terrace widths can be tuned by varying annealing conditions. Note that while roughness is similar in the unannealed substrate and the one unannealed at 1000°C, the former exhibits more irregular and meandering atomic steps compared to the latter.

To control the substrate morphology, *c*‐sapphire was annealed at 1000°C, 1200°C, and 1400°C for 12 h in the presence of air. A detailed surface characterization of both as‐received and annealed sapphire substrates was performed using atomic force microscopy (AFM) to evaluate the surface morphology (see Figure 1). Figure 1a shows the AFM image of the surface morphology for the as‐received sapphire substrate, Figure 1b for the sapphire substrate after film growth but without pre‐annealing (measured on parts of the sapphire substrates where no deposition occurred due to clamp shadowing), and Figure 1c–e for annealed sapphire under different conditions. The as‐received sapphire substrates exhibit high surface roughness, while thermal annealing significantly smooths the surface, leading to low‐roughness thermally equilibrated step‐terraced surfaces [35]. Previous reports suggest that the dimensions of atomic steps depend on both the annealing conditions and the impurity level of the sapphire substrate [35, 36, 37, 38].

Figure 1f–j shows the height profile of the atomic steps. In our case, annealing at 1000°C–1400°C results in increasing step heights and widths as a function of temperature. Further AFM statistical analysis reveals a clear evolution of the sapphire surface morphology with annealing temperature (Table S1). The unannealed and 1000°C‐annealed substrates exhibit low RMS roughness (*R_q_* = 0.64 – 0.70 Å), near‐zero skewness (*S_sk_* ≈ 0.01–0.02), and negative excess kurtosis values (*S_ku_* = −0.54 to −0.28), indicating smooth and symmetric terrace‐like morphologies. Already at the film growth conditions, small atomic steps are formed, although they are not as wide and straight as those observed during the dedicated annealing process (shown in Figure 1b). At 1200°C, the increased roughness (*R_q_* = 1.44 Å) together with slightly higher skewness and excess kurtosis values suggests surface restructuring. In contrast, the 1400°C‐annealed substrate shows significantly higher roughness (*R_q_* = 2.82 Å), large positive skewness (*S_sk_* = 1.12), and excess kurtosis (*S_ku_* = 3.28), indicating peak‐dominated surface features associated with enhanced step bunching and thermal surface reconstruction.

100 nm thin films of V_2_O_3_ were then grown on annealed or as‐received sapphire substrates using RF magnetron sputtering. ϕ‐scans for the $(20\bar{2}2$) peak of V_2_O_3_ showed three peaks at intervals of 120^○^, following the crystallographic orientation of the sapphire, revealing an epitaxial relationship between the films and substrates (Figure S3 and see also [28]). *θ‐2θ* X‐ray diffraction (XRD) scans reveal only the (000*l*) peaks of V_2_O_3_, alongside the sapphire peaks (Figure S4a). X‐ray reflectivity (XRR) measurements (Figure S4b,c) performed on V_2_O_3_ films grown on all sapphire substrates confirm that all films have identical thicknesses and densities up to the measurement resolution, further supporting the consistency in film quality.

**FIGURE 2:**
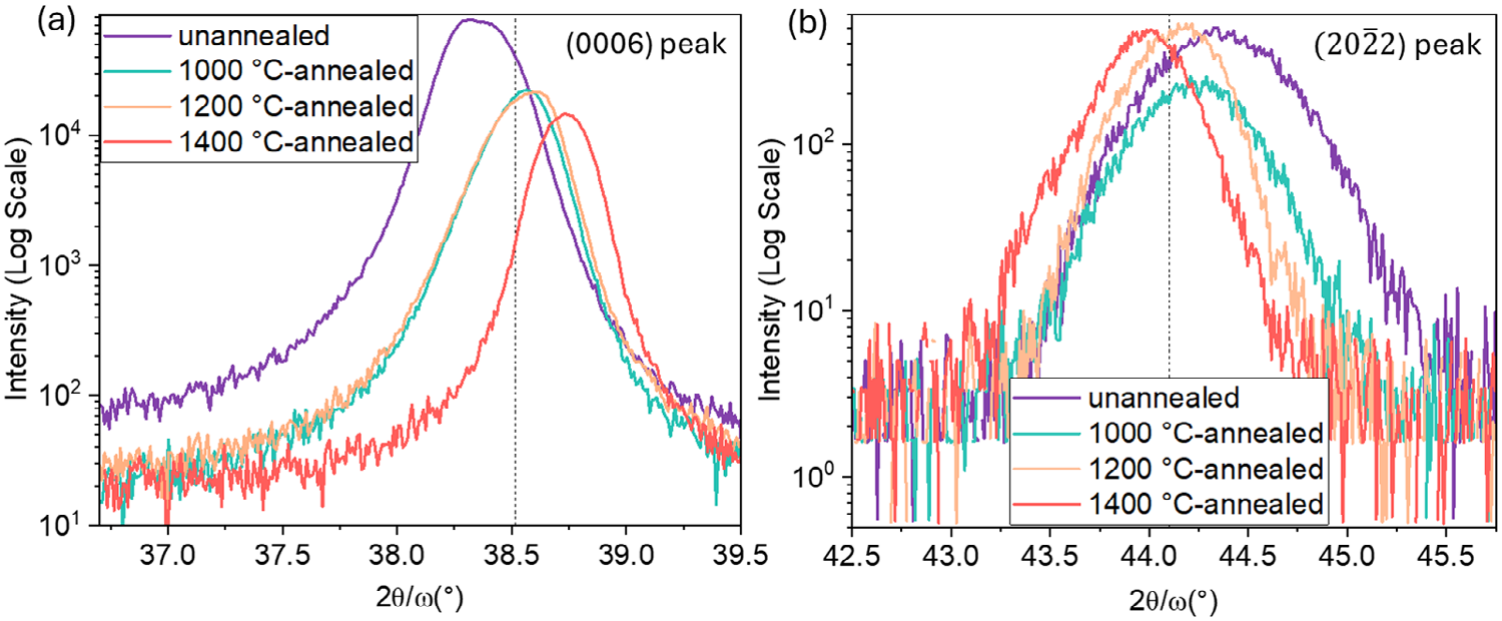
Reversal of strain regimes for V_2_O_3_ films grown on sapphire substrates with different annealing parameters: (a) out‐of‐plane 2θ/ω scan of the (0006) XRD peak for V_2_O_3_ films grown on sapphire substrates shows a systematic shift toward higher angles with increasing annealing temperature of the sapphire, indicating a transition from tensile to compressive strain consistent with out‐of‐plane lattice contraction. (b) The corresponding 2θ/ω scan of the $(20\bar{2}2$) peak shifts toward lower angles, further confirming strain relaxation in the V_2_O_3_ films due to annealing‐induced surface modifications of the sapphire. The dotted lines indicate the peak positions corresponding to bulk V_2_O_3_.

Lattice parameters and strain in the films were evaluated by 2θ/ω XRD scans on the $(20\bar{2}2$) and (0006) Bragg reflections shown in Figure 2 and Table S2. The V_2_O_3_ film grown on unannealed sapphire exhibits −0.54% ± 0.06% compressive strain in the *ab*‐plane and +0.48% ± 0.03% tensile strain along the *c*‐axis (Figure 2a,b) with respect to bulk V_2_O_3_. The asymmetric peak profile with a tail toward lower 2θ in the (0006) reflection indicates the presence of strain gradients along the *c*‐axis. With increasing substrate annealing temperature, a clear shift of the (0006) reflection peaks toward higher 2θ values is observed, surpassing the bulk value, signifying a transition from tensile to compressive strain in the *c*‐axis. (Figure 2a). Consistent with the Poisson effect, the $(20\bar{2}2$) reflection shifts from compressive to tensile strain as the substrate annealing temperature rises. The peak positions for the film grown on sapphire annealed at 1400°C are closer to those of the corresponding PI phase [29, 39, 40]. Additionally, with increasing substrate annealing temperature, the peaks become more symmetric, indicating a diminishing strain gradient within the films.

**FIGURE 3:**
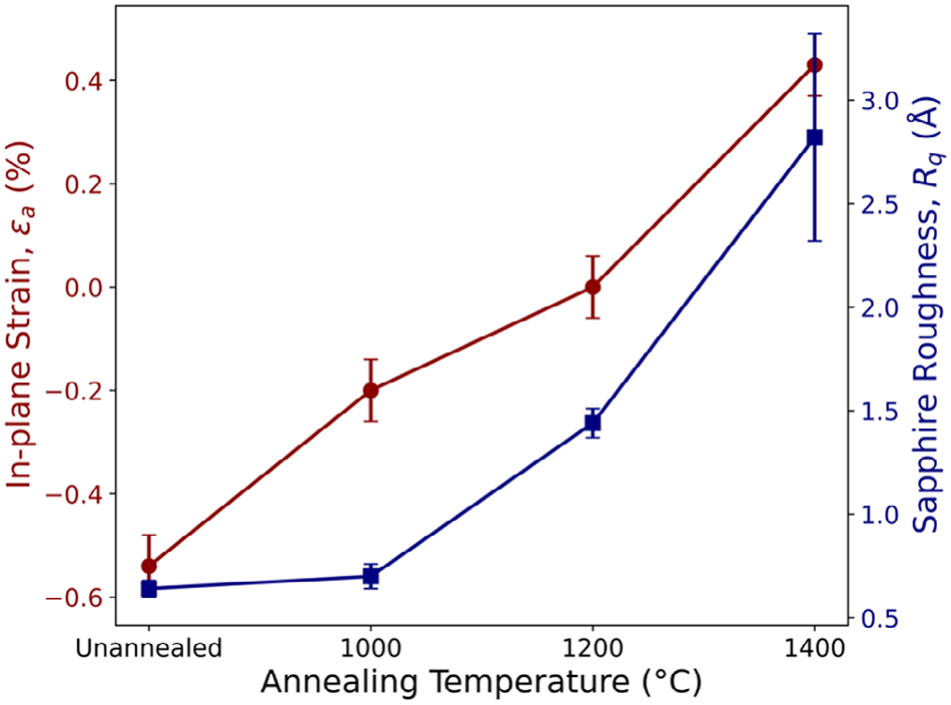
The effect of annealing sapphire substrates at different temperatures on their roughness (*R_q_*) and the in‐plane strain (*ε*_*a*_) of V_2_O_3_ films grown subsequently on the annealed substrates.

Figure 3 shows the evolution of the in‐plane strain (*ε*_*a*_) of the V_2_O_3_ films as a function of substrate annealing temperature together with the corresponding sapphire substrate roughness (*R_q_*). The results reveal a clear correlation between substrate morphology and strain evolution in the films. The in‐plane strain changes systematically from compressive strain for the unannealed substrate (−0.54% ± 0.06%) to a nearly relaxed state for the 1200°C‐annealed substrate (0.00% ± 0.06%), followed by tensile strain for the 1400°C‐annealed substrate (+0.43% ± 0.06%). Simultaneously, the sapphire surface roughness increases from 0.64 ± 0.04 Å for the unannealed substrate to 2.82 ± 0.50 Å after annealing at 1400°C. This trend suggests that the evolution of the substrate surface morphology strongly influences the strain relaxation behavior and the final residual strain state of the V_2_O_3_ films. Detailed statistical parameters of AFM measurements are summarized in Table S1, while the extracted lattice parameters and strain values are provided in Table S2.

In reciprocal space mapping (RSM) of the out‐of‐plane (0006) reflection, clear differences are observed between V_2_O_3_ films grown on unannealed and annealed *c*‐sapphire substrates (Figure S5). For films on unannealed sapphire, the (0006) peak is broad along Q_z_ and sharp along Q_x_, indicating significant strain gradients along the growth direction, while the in‐plane crystalline alignment remains relatively uniform. In contrast, films grown on annealed sapphire exhibit the opposite behavior: a sharp (0006) peak in Q_z_ and broadening in Q_x_, signifying relaxation in epitaxial alignment but the presence of in‐plane mosaic tilt due to defects, likely arising from step‐influenced growth or anisotropic relaxation at the terraced interface, as discussed below.

To measure the local strain distribution in the films and understand its relationship to the substrate morphology, high‐resolution scanning transmission electron microscopy (HRSTEM) was performed. Since the orientation along the steps is close to the $\left[\bar{1}010\right]$ crystallographic direction (see Figure 1e), lamellae were cut perpendicular to this orientation so that steps could be readily visualized. High‐angle annular dark field (HAADF) measurements were performed and interpreted using geometric phase analysis (GPA), allowing the extraction of relative local strains and the visualization of atomic planes and defects (see Figure S6). Two g‐vectors were used for the GPA analysis [41, 42] corresponding to the planes g_1_ = (0001) and g_2_ = $(\bar{1}2\bar{1}0$) (out‐of‐plane and in‐plane, respectively‐ see Figure S6b).

**FIGURE 4:**
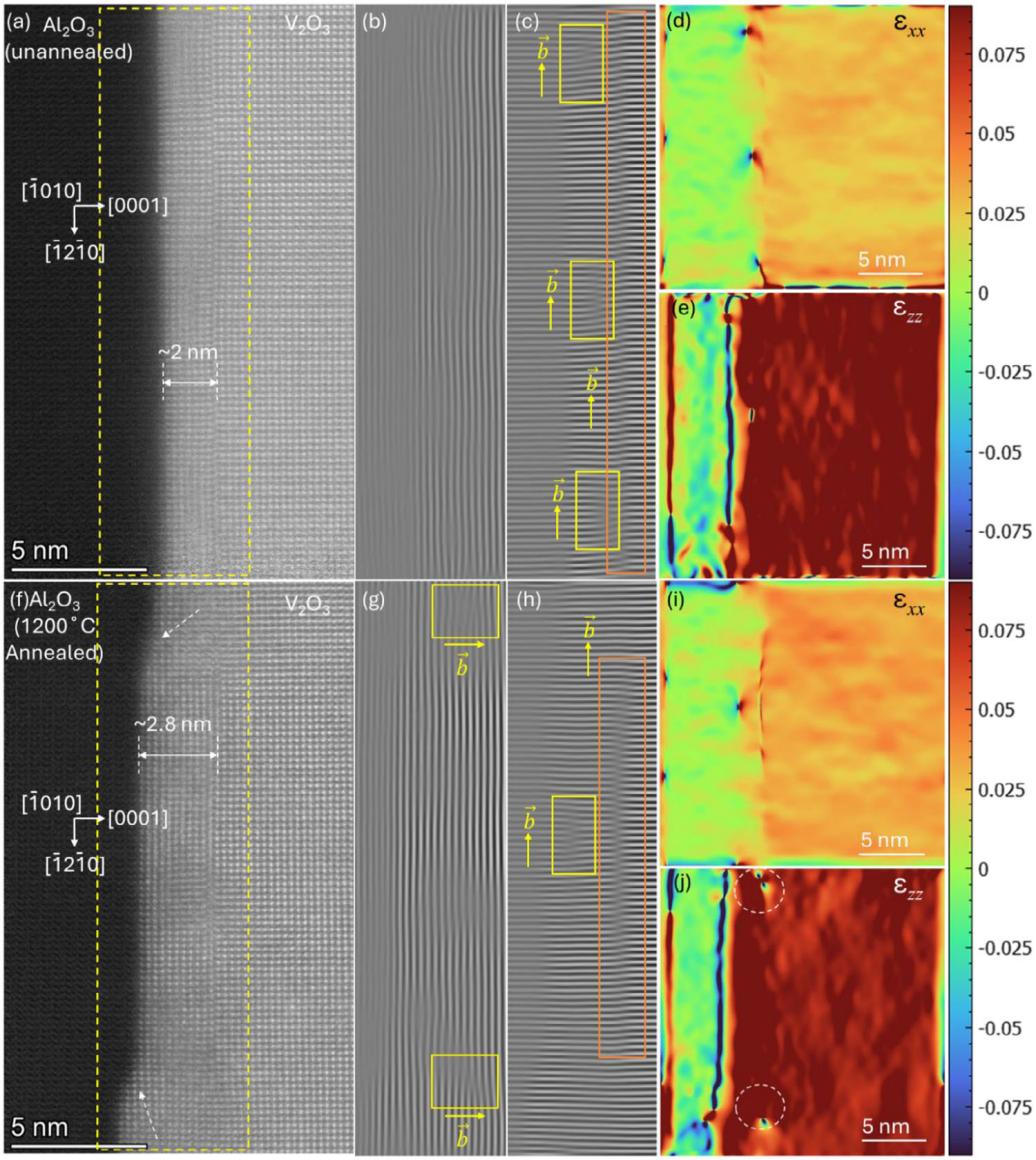
Imaging of dislocations and stacking faults and their effect on strain relaxation: (a) HAADF image of films on unannealed substrate; yellow dotted box shows region of interest (ROI), (b) (0001) Bragg‐filtered image of ROI which shows absence of partial dislocations with a Burgers vector perpendicular to the film plane. (c) Bragg‐filtered $(\bar{1}2\bar{1}0$) image exhibiting misfit dislocations (yellow rectangles) and a basal stacking fault (orange rectangle) extending beyond the scanned region. (d,e) In‐plane and out‐of‐plane components (respectively) of the strain tensor (ε_xx_ and ε_zz_) of the film grown on unannealed sapphire. (f) HAADF image of films on annealed sapphire at 1200°C. (g) (0001) Bragg‐filtered image of the ROI in (f) showing the presence of partial dislocations near steps with Burgers vectors along the [0001] orientation. (h) Bragg‐filtered $(\bar{1}2\bar{1}0$) image showing a stacking fault between the partial dislocations shown in (g). The Burgers vector derived from the circuit around this compound defect is parallel to the film's plane ($\vec{b}$ represents the Burgers vector direction with a magnitude of one atomic plane spacing). (i) and (j) In‐plane and out‐of‐plane components (respectively) of the strain tensor (ε_xx_ and ε_zz_) of the film grown on annealed sapphire at 1200°C.

Figure 4 compares V_2_O_3_ films grown on unannealed sapphire with those grown on sapphire annealed at 1200°C. Figure 4a presents HAADF‐STEM images of the substrate/film interface for films deposited on unannealed sapphire. The $(\bar{1}2\bar{1}0$) Bragg‐filtered image (Figure 4c) reveals misfit dislocations located very close to the interface, characterized by a missing $(\bar{1}2\bar{1}0$) atomic plane within the Burgers circuit ($\vec{b}$ represents the Burgers vector direction). A basal stacking fault is observed at a distance of ∼2 nm from the V_2_O_3_/sapphire interface and extends laterally beyond the scanned region. The stacking fault displacement vector has a dominant in‐plane component within the basal plane rather than a pure *c*‐axis shift, as it is invisible in the (0001) map but clearly visible in the in‐plane $(\bar{1}2\bar{1}0$) map. As shown in Figure 4d, the strain relaxes mostly due to the misfit dislocations close to the substrate/film interface and does not change appreciably between the two sides of the stacking fault. Therefore, most of the strain relaxation in this film occurs within the first 2 nm (Figure 4d,e). Previous reports also suggested V_2_O_3_ films grown on annealed sapphire relax after approximately ∼2–4 nm of growth [11, 13].

The sapphire substrate annealed at 1200°C exhibits two adjacent atomic steps, as indicated by the dotted arrows in Figure 4f. As in the case of the unannealed substrate, for the annealed substrate, the $(\bar{1}2\bar{1}0$) Bragg‐filtered images (Figure 4g,h) show misfit dislocations located very close to the interface, together with a stacking fault. However, in this film, the stacking fault is shorter and is terminated by partial dislocations, both of which lie ∼2.8 nm above the steps in the sapphire substrate. Furthermore, this stacking fault involves a missing atomic plane within the Burgers circuit that surrounds it (Figure 4h). Correspondingly, and in contrast to the unannealed substrate, an abrupt change in strain is observed when crossing the stacking fault; with increasing distance from the V_2_O_3_/sapphire interface, the in‐plane strain becomes less compressive (while the out‐of‐plane strain becomes less tensile, consistent with the Poisson effect). This compound defect can be regarded as an extended misfit dislocation which has dissociated into two partial dislocations and a stacking fault. The presence of the SF boundaries just on top of the sapphire atomic steps suggests that the steps reduce the energy to form the partial dislocations at the SF boundary. Thus, the terraced morphology of the substrate is found to be more conducive to strain relaxation by defects compared to the flat unannealed substrate.

**FIGURE 5:**
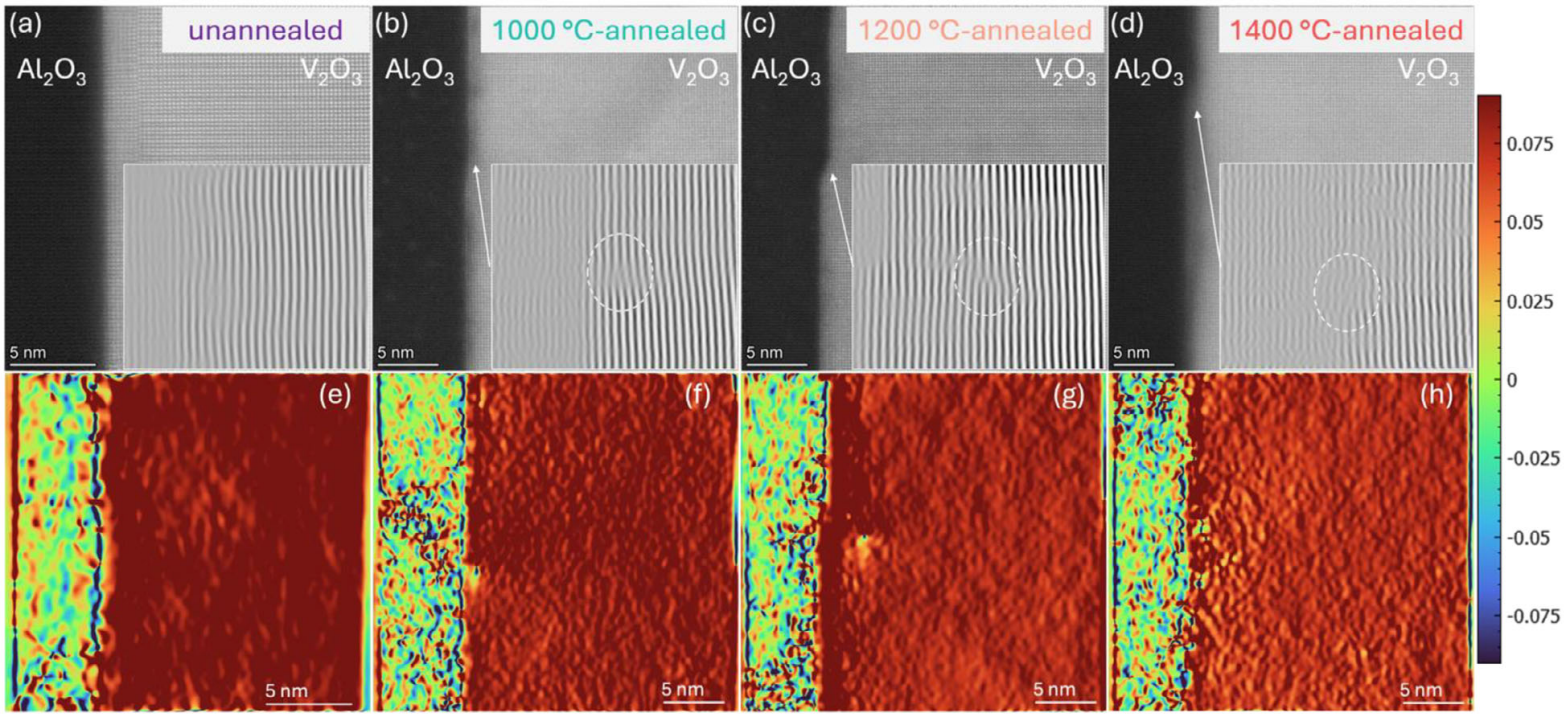
Effect of substrate annealing at different temperatures on strain relaxation through formation of dislocations in V_2_O_3_ films: (a–d) HRSTEM images showing atomic steps on the surface of annealed (0001)‐sapphire, which are absent in unannealed sapphire. The step heights correspond to sub‐unit cell dimensions. Inset: (0001) Bragg‐filtered image highlighting localized defects near the atomic steps in the sapphire substrate. (e–h) Out‐of‐plane components of the strain tensor reveal increasing tensile strain with increasing sapphire annealing temperature. These sub‐unit‐cell steps, as well as roughness and associated defects, promote strain relaxation in V_2_O_3_ films grown on (0001)‐sapphire, facilitating partial accommodation of lattice mismatch and reducing in‐plane strain.

The same GPA analysis conducted for films annealed at different temperatures (Figure 5) reveals that the increased substrate roughness induced by annealing results in a higher defect density in the V_2_O_3_ film, which is more effective in relaxing compressive strain in the vicinity of the substrate. For instance, the sample annealed at the maximum temperature (1400 C) has the largest roughness and also the largest *a* lattice parameter. As shown in Figure 5d,h, in addition to a step, for this sample the substrate/film interface exhibits a region of intermediate contrast, in accordance with the increased roughness within the terrace as found in the AFM topography (Figure 1e). This sample exhibits a high density of defects near the interface and a larger and more abrupt strain relaxation compared with the samples where the substrate was annealed at a lower temperature (Figure 5a–c,e–g).

We note that surface roughness is not the only parameter governing strain relaxation. For example, the unannealed substrate and the substrate annealed at 1000°C exhibit similarly low roughness values (0.64–0.7 Å, see Figure 1b,c; Table S1). Nevertheless, the *V*_2_ *O*_3_ films grown on the annealed substrate display significantly greater strain relaxation than those grown on the unannealed substrate.

This difference can be understood in terms of the strain‐relaxation mechanism associated with dislocation formation near atomic steps. Although the two substrates exhibit comparable roughness, their step morphologies differ noticeably: the steps on the annealed substrate are more regular and less meandering than those on the unannealed substrate. Consequently, the energy cost for forming dislocations near straighter step edges is expected to be lower, thereby promoting more efficient strain relaxation in the annealed sample, consistent with our observations.

The HRSTEM results are fully consistent with the average strain measured by XRD (Table S2), showing that with increasing substrate annealing temperature the *a* lattice parameter increases and the *c* parameter decreases. The reason for the transition from compressive to tensile in‐plane strain (and vice‐versa for out‐of‐plane strain) is related to the difference in thermal contraction between the substrate and film, and is considered in detail in the Discussion section.

**FIGURE 6:**
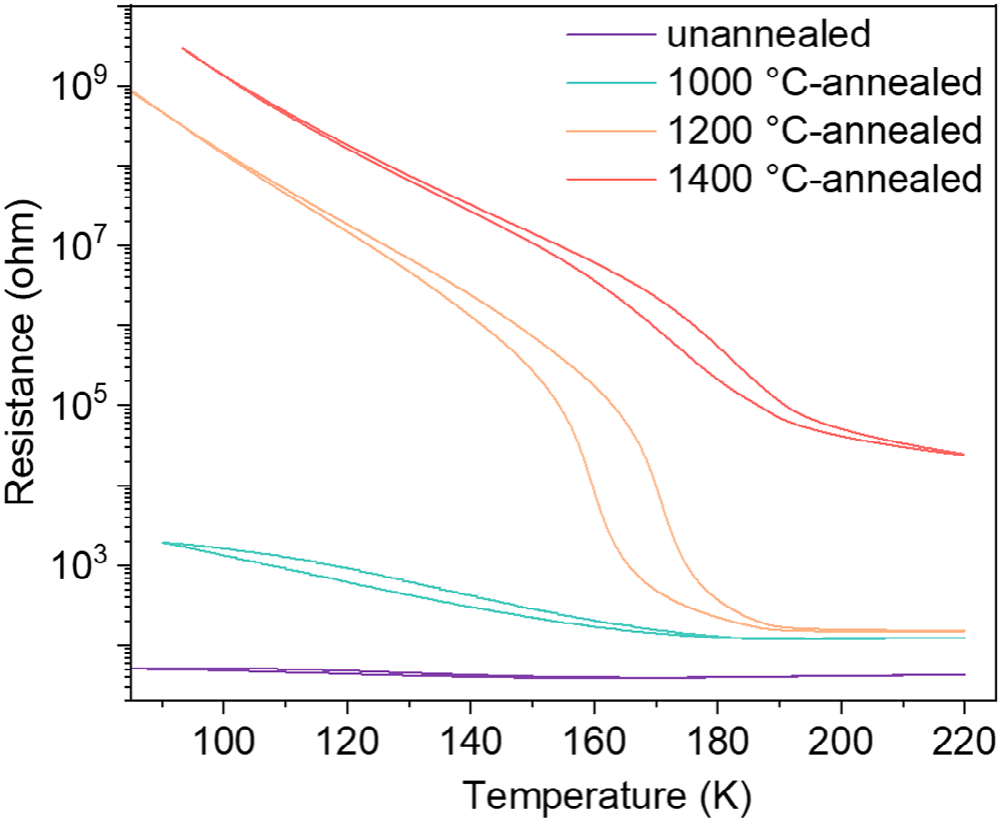
Temperature‐dependent resistance of V_2_O_3_ films grown on sapphire substrates annealed at different temperatures: The film on unannealed sapphire shows a suppressed MIT, while films on sapphire annealed at 1000°C and 1200°C display progressively larger resistive transitions, with the 1200°C film exhibiting a six‐order resistance change. At 1400°C, a PI phase emerges above 200 K and exhibits a PI‐AFI transition.

The morphology‐driven variations in film strain lead to a drastic change in the electronic properties of the films. In Figure 6, the R(T) measurements reveal significant differences in the transport behavior of V_2_O_3_ films depending on the sapphire substrate treatment. The film grown on unannealed sapphire exhibits a strongly suppressed MIT. In contrast, the film grown on sapphire annealed at 1000°C shows a partial transition, with a resistance change of approximately one order of magnitude. Films grown on substrates annealed at 1200°C display a fully developed MIT, with a resistance change of six orders of magnitude at the transition temperature of 160 K. The film grown on sapphire annealed at 1400°C displays a negative *dR/dT* and nearly three orders of magnitude higher resistance above 200 K compared to the film grown on the unannealed sapphire. This behavior is consistent with the PI phase of V_2_O_3_, which is known to arise for large in‐plane tensile strain [29, 32, 43].

It is also noteworthy that the duration of annealing plays a crucial role in determining the dimensions of atomic steps on the substrate surface. As illustrated in Figure S7a–c, annealing sapphire at 1000°C for 6 h results in relatively small atomic steps, whereas extending the annealing time to 24 h leads to the formation of larger steps. These enlarged steps increase the likelihood of defect formation in the V_2_O_3_ films, which facilitates strain relaxation, thereby strongly affecting transport properties (Figure S7d).

## 3 Discussion

XRD measurements of the various V_2_O_3_ films grown on (0001)‐oriented sapphire substrates reveal a systematic evolution of the strain state with substrate annealing temperature. The largest compressive in‐plane strain is found for films grown on substrates that were not annealed prior to the growth. With increasing annealing temperature and duration, the compressive strain relaxes and eventually changes sign to become tensile.

To understand the microscopic origin of this strain evolution, AFM measurements of the substrates were performed, which revealed that substrate annealing produces a stepped morphology and increased roughness. Subsequently, HRSTEM measurements were performed on the V_2_O_3_ films, focusing on the film‐substrate interface. These measurements revealed strain‐relaxation pathways, particularly through the formation of defects around the atomic steps and rough regions in the sapphire substrate. The combined HRSTEM and XRD results therefore provide complementary insight into both the local relaxation mechanisms and the global residual strain state of the films.

However, the relaxation mechanisms revealed by HRSTEM alone cannot explain the in‐plane *tensile* strain observed in films grown on substrates annealed at 1400°C. An additional contribution arises from the thermal expansion mismatch between sapphire and V_2_O_3_. Since the films are grown at temperatures of approximately 1000 K, whereas the rich phase diagram of V_2_O_3_ occurs below 400 K, thermal contraction during cooling is expected to play an important role in determining both the final strain state and the phase stability of the films.

**FIGURE 7:**
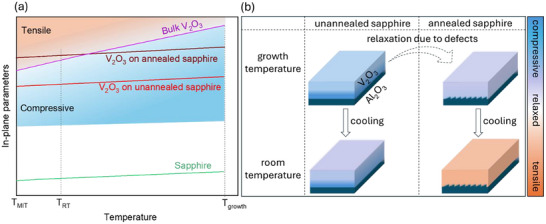
Mechanism for formation of compressive/tensile in‐plane strain in V_2_O_3_ films: (a) In‐plane lattice parameters of bulk V_2_O_3_, V_2_O_3_ film, and sapphire as a function of temperature. At the growth temperature, the in‐plane lattice parameters of the films will range between those of bulk V_2_O_3_ and sapphire depending on the extent of compressive strain relaxation, as determined by the substrate morphology. Due to clamping, the film's in‐plane lattice contraction (a(T)‐curve slope) follows that of the sapphire rather than that of bulk V_2_O_3_. Depending on the a‐parameter achieved during growth, film/bulk parameters may or may not intersect upon cooling, resulting in tensile or compressive strain in the film at low T, respectively. (b) Schematics of the effect of the terrace morphology on the strain during growth and upon cooldown. For unannealed substrates, large in‐plane compressive strain develops in the film due to the lattice mismatch and partially decreases due to thermal expansion mismatch. When grown on annealed sapphire, atomic steps in the substrate facilitate the formation of dislocations and stacking faults in the film, which reduce the compressive strain at the growth temperature. After cooldown, the thermal expansion mismatch produces in‐plane tensile strain in the film.

To examine this effect, we performed temperature‐dependent XRD measurements of the $(11\bar{2}0$) peak between 200 and 600 K (limited by our experimental setup), from which we extracted the *a* lattice parameter of the film (see Figure S8). Rather than following the thermal expansion behavior of bulk V_2_O_3_, the film lattice parameter evolves similarly to sapphire, indicating strong substrate clamping. Figure 7a schematically illustrates the effect of this clamping on the strain evolution. At the growth temperature, the in‐plane lattice parameter of the film lies between that of bulk V_2_O_3_ and sapphire, depending on the degree of strain relaxation achieved during growth. Upon cooling, the *a* lattice parameter of the film contracts less strongly than in bulk V_2_O_3_ due to the clamping effect of the substrate. Consequently, the compressive in‐plane strain decreases during cooling and may even become tensile if the film *a* lattice parameter exceeds that of bulk V_2_O_3_.

The strain state established at the growth temperature therefore determines whether the film becomes tensile after cooling. Greater relaxation during growth leads to a more tensile strain state at low temperature, whereas more moderate relaxation leaves the film strongly compressively strained despite the partial compensation arising from thermal contraction mismatch.

As demonstrated in this study, substrate morphology is the key parameter controlling the extent of relaxation, by promoting the formation of misfit dislocations and stacking faults (Figure 7b). Assuming perfect clamping below the growth temperature, the thermal expansion mismatch alone would generate an additional tensile in‐plane strain of approximately 0.74% at room temperature [33, 34]. Using this estimate, the in‐plane strain at the growth temperature is inferred to range from − 1.28% for films grown on unannealed substrates to − 0.31% for films grown on substrates annealed at 1400°C. Compared to the nominal lattice mismatch of − 4.1%, these values indicate that substantial strain relaxation already occurs even for substrates with relatively low roughness.

In terms of transport properties, the substrate‐induced strain variations have a drastic effect. A nearly complete suppression of the resistive transition is observed in the most compressively strained films. In contrast, the most tensile‐strained films exhibit stabilization of the PI phase at room temperature, with the transition to the AFI phase occurring only near ∼ 180K. It is worth noting that small variations in the V:O ratio in bulk V_2_O_3+x_ are known to reduce the PM–AFI transition temperature, which bears some similarity to the present observations [44]. However, all films in this study were grown simultaneously under identical conditions, making significant stoichiometric variations unlikely. Furthermore, the PI phase has not been reported in the literature as arising from variations in the V:O ratio in bulk V_2_O_3+x_, in contrast to the insulating phase observed above 200 K in the most tensile‐strained sample. Although minor stoichiometric variations cannot be completely excluded, they are therefore unlikely to be responsible for the observed behavior.

Overall, these results provide direct microscopic evidence linking substrate morphology, strain relaxation, thermal expansion mismatch, and the resulting electronic transport properties of V_2_O_3_ thin films.

## 4 Conclusions

In summary, this study demonstrates that annealing sapphire substrates induces tuneable atomically stepped terraces, which play a pivotal role in modulating strain and transport properties in V_2_O_3_ thin films. High‐resolution STEM data reveal that films grown on annealed sapphire exhibit structural defects that serve as relaxation pathways for compressive strain. Upon cooldown, clamping to the substrate reduces the in‐plane compressive strain and may even result in tensile strain, depending on the extent of relaxation of the compressive strain during growth. Thus, minute changes in substrate morphology may induce reversal of the strain state in the films at low temperature, ranging from compressive to tensile states. AFM, transport and XRD data confirm this scenario and show that controlling substrate morphology can induce drastic changes in film properties. These range from stabilization of the metallic phase at all temperatures (strong compressive strain), to a fully developed metal‐insulator transition (nearly relaxed), to fully insulating at all temperatures (strong tensile strain). Overall, atomic step‐mediated strain engineering offers a powerful strategy to control strain and phase transitions and may be broadly applicable to other oxides and Mott materials. This strategy provides a pathway to tune electronic phase transitions for next‐generation resistive switching applications.

## Conflicts of Interest

The authors declare no conflicts of interest.

## Supporting information




**Supporting File**: smll74217‐sup‐0001‐SuppMat.docx.

## Data Availability

The data that support the findings of this study are openly available in Zenodo repository using the following: https://doi.org/10.5281/zenodo.20743858.
