# Supplementary material for: Reversal of Strain State in a Mott Insulator Thin Film by Controlling Substrate Morphology
Source: Small. 2026 Jun 23;22(45):e74217. doi: 10.1002/smll.74217 (PMC13472277; doi:10.1002/smll.74217)
Supplement: Supplementary file 1 [file SMLL-22-e74217-s001.docx]

Supplementary information

**Reversal of Strain State in a Mott Insulator Thin Film by Controlling Substrate Morphology**

Reetendra Singh^1^, Abhishek Rakshit^1^, Galit Atiya^1^, Michael Kalina^1^, Yaron Kauffmann^1^, and Yoav Kalcheim^1^*

^1^Department of Materials Science and Engineering, Technion-Israel Institute of Technology, Haifa 32000, Israel

*Email: ykalcheim@technion.ac.il

**Experimental methods**

Substrate thermal treatment and morphology measurements:

(0001)*-*sapphire substrates from MTI Corporation were annealed in air at 1000 °C, 1200 °C, and 1400 °C using a Kejia furnace. The morphology of the substrates was characterized by Atomic Force Microscopy (AFM). The dimensions of atomic steps on the sapphire surface were determined from surface roughness measurements using Gwyddion software.

Growth of V_2_O_3_ thin films:

100 nm V_2_O_3_ films were grown on as-received, unannealed and annealed sapphire substrates of by RF magnetron sputtering in 5 mTorr Ar atmosphere and at a substrate temperature of 750 °C. Following growth, the samples were rapidly thermally quenched^[1]^.

Structural characterization of the films:

To determine the lattice constants of the V_2_O_3_ films, we first aligned the symmetric (0006) and asymmetric $(20\bar{2}2$) reflections using a Rigaku Smartlab diffractometer equipped with a four-circle goniometer. The sample stage allows independent rotations about two perpendicular axes (R_x_ and R_y_), azimuthal rotation (φ), and tilt (χ) in the diffraction plane. Measurements were performed using Cu Kα₁ radiation (λ = 1.540593 Å). The (0006) peak, corresponding to the out-of-plane c-axis lattice spacing, was aligned in a symmetric ω-2θ geometry. After coarse alignment on a strong substrate peak, we performed a high-resolution ω-2θ scan around the nominal position of the (0006) reflection. The peak position was refined by adjusting sample height and tilt until maximum intensity was achieved.

The *c*-parameter was extracted using the formula relating (*hkl*) to d-spacing for a hexagonal crystal:

$\frac{1}{d^{2}}=\frac{4}{3}\left( \frac{h^{2}+hk+k^{2}}{a^{2}} \right)+\frac{l^{2}}{c^{2}}$	(1)

and Bragg's law: nλ = 2*d* sinθ	(2)

λ is the wavelength of X-rays (Cu K_α_= 1.5406 Å).

The in-plane strain is ε*_a_* (%)$=\left[ \frac{a_{(film)}-a_{(bulk)}}{a_{(bulk)}} \right]$x100	(3)

and out-of-plane strain ε*_c_* (%) $=\left[ \frac{c_{(film)}-c_{(bulk)}}{c_{(bulk)}} \right]$x100	(4)

Errors of in-plane and out-of-plane strains:

$\Delta$ε*_a_* (%)$=\left[ \frac{\Delta a}{a_{(bulk)}} \right]$x100	(5)

$\Delta$ε*_c_* (%) $=\left[ \frac{\Delta c}{c_{(bulk)}} \right]$x100	(6)

Using equations (1)-(2) and the measured (0006) and (202̅2) peak positions, the film's *c*- and *a*-axis lattice constants were determined. Strains and corresponding errors were calculated using equations (3)-(6) and shown in **Table 1** in the main text. The bulk V₂O₃ lattice parameters *a*_(_*_bulk_*_)_ and *c*_(_*_bulk_*_)_ were taken from ^[2]^ to calculate in-plane and out-of-plane strains and their errors.

Fits to XRR measurements were performed using the differential evolution algorithm GenX ^[3]^.

Transport measurements:

Electrical transport measurements were performed using a Lakeshore TTPX probe station equipped with a Keithley 2450 source meter unit. Probes were placed on indium contacts soldered on the V_2_O_3_ samples.

HRSTEM:

Lamellas were fabricated along the $[\bar{1}010]$ direction of sapphire from a 100 nm thick V_2_O_3_ film using a Thermo Fisher Helios 5 Plasma Focused Ion Beam (PFIB).

High-resolution STEM imaging was performed on a Thermo Fisher/FEI double corrected Titan Themis TEM, operated at 300 keV and using the HAADF-STEM detector. Atomic resolution STEM micrographs were acquired using averaged multiple fast acquisition frames to avoid any scan distortion for reliable GPA analysis.

**Supplementary figures**

**Supplementary figure 1.** Resistance versus temperature for a 100 nm V_2_O_3_ film grown on sapphire substrates with different orientations. Films on *c*-sapphire shows a strongly suppressed metal-insulator transition, whereas films grown on other sapphire orientations exhibit a full transition of 5-7 orders of magnitude. The inset presents the resistance–temperature curve of V_2_O_3_ films grown on (0001)-sapphire under similar conditions. Under substrate-induced 2D confinement, V_2_O_3_ film on (0001)-sapphire faces restricted in-plane (*ab*-plane) expansion, necessary for the transition to the monoclinic phase. In other orientations, out-of-plane expansion allows for this distortion.

**Supplementary figure 2.** (a) Graphical illustration showing that during the MIT, the monoclinic distortion causes the [0001] direction to contract while the perpendicular directions expand. (b) In (0001)-oriented growth, the lattice expansion is restricted to the film plane, so the monoclinic transition increases in-plane compressive stress; in contrast, other orientations allow out-of-plane expansion, thereby enabling the monoclinic distortion.

| **Sample** | ***R_q_* (**Å) | **Skewness (*S_sk_*)** | **Excess kurtosis** |
| --- | --- | --- | --- |
| as-received | 2.68 ± 0.50 | 1.29 ± 0.49 | 2.44 ± 1.91 |
| unannealed | 0.64 ± 0.04 | 0.01 ± 0.16 | -0.54 ± 0.26 |
| 1000 °C-annealed | 0.70 ± 0.06 | 0.02 ± 0.21 | -0.28 ± 0.34 |
| 1200 °C-annealed | 1.44 ± 0.07 | 0.03 ± 0.14 | -0.62 ± 0.21 |
| 1400 °C-annealed | 2.82 ± 0.50 | 1.12 ± 0.60 | 3.28 ± 2.47 |

**Supplementary Table 1.** AFM statistical analysis reveals a strong evolution of the surface morphology with annealing temperature. The unannealed and 1000 °C-annealed sapphire exhibit low RMS roughness (*R_q_*) together with near-zero skewness (*S_sk_*) and negative kurtosis values, indicating smooth and symmetric terrace-like surface morphologies without pronounced surface protrusions. In the 1200 °C-annealed sapphire, the values of roughness, skewness and kurtosis increased suggesting surface restructuring.1400 °C-annealed substrate exhibits significantly increased roughness along with large positive skewness and kurtosis values, indicating the formation of peak-dominated and sharply varying surface features, likely associated with enhanced step bunching and thermal surface reconstruction at elevated temperatures.

**Supplementary figure 3.** 𝜙-scans for the $(20\bar{2}2$) peak of (a) sapphire and V_2_O_3_ on unannealed sapphire and (b) V_2_O_3_ on annealed sapphire at 1200°C showed three peaks at intervals of 120°, following the crystallographic orientation of the sapphire, revealing an epitaxial relationship between the films and substrates.

**Supplementary figure 4.** (a) XRD patterns of V_2_O_3_ films grown on (0001)-sapphire substrates annealed under different conditions confirming that up to strain, all samples show the same structure. (b) XRR and (c) its first derivative for V_2_O_3_ films grown on (0001)-sapphire with varying substrate-annealing conditions, confirming similar film quality, thickness and density across all samples.

**Supplementary Table 2.** Annealing conditions of sapphire, lattice parameters, and the corresponding in-plane and out-of-plane strain within the films.

**Supplementary figure 5.** The RSM of the (0006) reflection for films grown on (a) unannealed sapphire and (b) annealed sapphire at 1000°C shows that films on unannealed sapphire exhibit a strain gradient along the c-axis due to substrate clamping. This strain is relieved in the films grown on annealed sapphire, resulting in film tilting and disordering.

**Supplementary figure 6**. (a) HAADF image showing two adjacent atomic steps on sapphire annealed at 1200 °C (see dotted circles). (b) power spectrum of the image and g-vectors g_1_=(0001) and g_2_=$(\bar{1}2\bar{1}0$).

Geometric phase analysis (GPA) was carried out using the *Strain ++* open-source software package ^[4]^. The two intense reflections in the power spectrum, g_1_=(0001) and g_2_=$(\bar{1}2\bar{1}0$), were selected to compute the displacement field, enabling the generation of local strain maps and filtered Bragg images for defect analysis.

**Supplementary figure 7.** (a, b) AFM images of annealed (0001)-sapphire showing atomic steps; (c) corresponding line profiles indicating the step height and width for substrates annealed at 1000 °C for 6 h and 24 h, respectively; (d) temperature-dependent resistance of V_2_O_3_ films grown on these annealed sapphire substrates.

**Supplementary figure 8.** In-plane lattice parameters of bulk V_2_O_3_, V_2_O_3_ film, and sapphire as a function of temperature. At the growth temperature the in-plane lattice parameters of the films will range between those of bulk V_2_O_3_ and sapphire depending on the extent of compressive strain relaxation, as determined by the substrate morphology. Due to clamping, the film in-plane lattice contraction follows that of the sapphire rather than that of bulk V_2_O_3_. Upon cooldown the film/bulk parameters may or may not intersect, resulting in tensile or compressive strain in the film at low T, respectively. The *a*-parameters for sapphire and bulk V_2_O_3_ were taken from the previous reports ^[5-7]^.

**Supplementary references**

1. Trastoy, J.; Kalcheim, Y.; Del Valle, J.; Valmianski, I.; Schuller, I. K. Enhanced metal–insulator transition in V_2_O_3_ by thermal quenching after growth. *J. Mater. Sci.* **2018**, *53*(12), 9131-9137.
2. Grygiel C.; Simon C.; Mercey B.; Prellier W.; Fresard R.; Limelette P. Thickness dependence of the electronic properties in V_2_O_3_ thin films. *Appl. Phys. Lett.* **2007**, 91, 262103.
3. Glavic, A.; Björck, M. GenX 3: the latest generation of an established tool. *J. Appl. Cryst.* **2022**, 55, 1063-1071.
4. Hÿtch, M. J.; Snoeck, E.; Kilaas, R. Quantitative measurement of displacement and strain fields from HREM micrographs. *Ultramicroscopy* **1998**, 74, 131-146.
5. Roder, C.; Einfeldt, S.; Figge, S.; Paskova, T.; Hommel, D.; Paskov, P. P.; Monemar, B.; Behn, U.; Haskell, B. A.; Fini, P. T.; Nakamura, S. Stress and wafer bending of *a*-plane GaN layers on *r*-plane sapphire substrates. *J. Appl. Phys.* **2006**, 100, 103511.
6. Belbéoch, B.; Kleinberger, R.; Roulliay, M. Lattice parameter anomalies in V_2_O_3_ at high temperature. *Solid State Commun.* **1978**, 25, 1043.
7. Eckert, L. J.; Bradt, R. C. Thermal expansion of corundum structure Ti_2_O_3_ and V_2_O_3_. *J. Appl. Phys.* **1973**, 44, 3470.
